# Daily longitudinal self-monitoring of mood variability in bipolar disorder and borderline personality disorder

**DOI:** 10.1016/j.jad.2016.06.065

**Published:** 2016-11-15

**Authors:** A. Tsanas, K.E.A. Saunders, A.C. Bilderbeck, N. Palmius, M. Osipov, G.D. Clifford, G.Μ. Goodwin, M. De Vos

**Affiliations:** a Institute of Biomedical Engineering, Department of Engineering Science, University of Oxford, UK; b Oxford Centre for Industrial and Applied Mathematics, Mathematical Institute, University of Oxford, UK; c Sleep and Circadian Neuroscience Institute, Nuffield Department of Clinical Neurosciences, UK; d Department of Psychiatry, University of Oxford, UK; e Department of Biomedical Informatics, Emory University, Atlanta, GA, USA; f Department of Biomedical Engineering, Georgia Institute of Technology, USA

**Keywords:** Bipolar disorder, Borderline personality disorder, Digital health, Mood assessment, Mood monitoring, Patient reported outcome measures

## Abstract

**Background:**

Traditionally, assessment of psychiatric symptoms has been relying on their retrospective report to a trained interviewer. The emergence of smartphones facilitates passive sensor-based monitoring and active real-time monitoring through time-stamped prompts; however there are few validated self-report measures designed for this purpose.

**Methods:**

We introduce a novel, compact questionnaire, Mood Zoom (MZ), embedded in a customised smart-phone application. MZ asks participants to rate anxiety, elation, sadness, anger, irritability and energy on a 7-point Likert scale. For comparison, we used four standard clinical questionnaires administered to participants weekly to quantify mania (ASRM), depression (QIDS), anxiety (GAD-7), and quality of life (EQ-5D). We monitored 48 Bipolar Disorder (BD), 31 Borderline Personality Disorders (BPD) and 51 Healthy control (HC) participants to study longitudinal (median±iqr: 313±194 days) variation and differences of mood traits by exploring the data using diverse time-series tools.

**Results:**

MZ correlated well (|R|>0.5,p<0.0001) with QIDS, GAD-7, and EQ-5D. We found statistically strong (|R|>0.3,p<0.0001) differences in variability in all questionnaires for the three cohorts. Compared to HC, BD and BPD participants exhibit different trends and variability, and on average had higher self-reported scores in mania, depression, and anxiety, and lower quality of life. In particular, analysis of MZ variability can differentiate BD and BPD which was not hitherto possible using the weekly questionnaires.

**Limitations:**

All reported scores rely on self-assessment; there is a lack of ongoing clinical assessment by experts to validate the findings.

**Conclusions:**

MZ could be used for efficient, long-term, effective daily monitoring of mood instability in clinical psychiatric practice.

## Highlights

• Introducing a novel compact questionnaire called Mood Zoom (MZ) which expedites daily mood monitoring.
• MZ comprises six items on a 7-point Likert scale, and easily fits a smartphone screen.
• MZ correlated well against established clinical questionnaires.
• MZ can differentiate bipolar disorders and borderline personality disorders.

## 1 Introduction

The potential benefits of reliable monitoring of symptom severity is acknowledged in many chronic conditions (Steventon et al., 2012, Tsanas, 2012), but particularly for mental health (American Psychiatric Association, 2013; Holmes et al., 2016, Lanata et al., 2015, Solomon et al., 2010). Residual symptoms are important in psychiatric disorders because they directly impair social and economic activity and increase the risk of new episodes. Capture and monitoring of symptom variability and progression prospectively (Slade, 2002, Solomon et al., 2010) is accordingly widely encouraged in treatment guidelines.

Monitoring of mood states is often used in the assessment and management of mood disorders. Traditionally, self-monitoring of mood using Patient Reported Outcome Measures (PROMs) was achieved using paper-based and more recently computer-based questionnaires (Bopp, 2010, Malik, 2012) but in recent years the ubiquity of mobile networks and the rapid evolution of smartphone technology have led to an increasing focus on the use of mobile applications (Faurholt-Jepsen et al., 2015, Schärer et al., 2015, Schwartz et al., 2016). This approach has advantages because mood states can be reported in real time without the inconvenience of logging to a computer and thus self-ratings should be less prone to recall bias (Proudfoot et al., 2010). However, the optimal temporal frequency of mood monitoring remains the source of some uncertainty (Moore et al., 2014). Here, we describe the validation of a smartphone-based application for the delivery of daily mood monitoring in two patient groups where mood instability is a common. Bipolar Disorder (BD) and Borderline Personality Disorder (BPD) affect around 2% of the population respectively. Traditional descriptions of BD comprising clear episodes of elated or depressed mood interspersed with periods of euthymia mask the true course of the disorder which is characterised by chronic mood instability and poor inter-episode function. The duration of these periods may vary considerably from weeks to months, with depression typically dominating the longitudinal course of the disorder (Anderson et al., 2012). Borderline personality disorder is a pervasive disorder where mood instability is accompanied by impulsivity, interpersonal dysfunction, repeated suicidal gestures, an uncertain sense of self, inappropriate anger and a fear of abandonment. Mood instability in BPD is thought to differ from other disorders in its nature (Koenigsberg et al., 2002) and relate to an inability to modulate emotional responses (Gratz et al., 2006, Linehan, 1993) although few direct comparisons with BD have been made. BD and BPD can be clearly distinguished using laboratory measures of social cooperation and reward learning (Saunders et al., 2015) but in clinical practice their distinction can be far more challenging. Correct diagnosis is essential given their divergent treatment approaches; BD requires a long term medication (Goodwin et al., 2016) whereas there are no licensed medications for BPD and psychological interventions are recommended (NICE, 2009). We stress that this study focuses on mood variability and not emotional dysregulation. The latter refers to short-term (from seconds to a few hours) behavioural outbursts, and is the result of poor regulation of emotional responses. Mood is less specific than emotions and refers to an internal psychological state which can last from hours to months; mood variability aims to characterize long-term mood disturbances.

The aims of the study were to: (a) introduce and validate a novel clinical questionnaire used for *daily* mood monitoring as part of a smartphone application, (b) explore the longitudinal variation in mood characteristics of BD, BPD, and Healthy Control (HC) participants extracted from this new questionnaire as compared to four established psychiatric questionnaires quantifying mood on a *weekly* basis and (c) to test the hypothesis that mood variability might discriminate BD and BPD groups from HC and more critically from each other. We present results from a relatively large number of participants in the context of longitudinal mood monitoring, tracking their mood variation for *multiple months*, as opposed to other studies that were confined to a *few weeks* (e.g. Holmes et al., 2016, Schwartz et al., 2016), and using multiple questionnaires (most previous studies focus on a single questionnaire to investigate symptom variation, e.g. depression, for example Bonsall et al., 2012, Moore et al., 2014, Bonsall et al., 2015, Holmes et al., 2016). Moreover, most other studies focus solely on a single disorder (e.g. BD, Bonsall et al., 2015, Faurholt-Jepsen et al., 2015, Holmes et al., 2016, Lanata et al., 2015), whereas we have also recruited people diagnosed with BPD, and compared findings against HC.

## 2 Data

The data were collected as part of the Automated Monitoring of Symptom Severity (AMoSS) study exploring mood, activity and physiological variables (Palmius et al., 2014). The study was observational, and independent from the clinical care the participants received. We recruited 139 participants: 53 diagnosed with BD, 33 diagnosed with BPD and 53 age-matched HC. BD and HC were also gender-matched; the BPD group were predominantly female. The participants were recruited for an initial three-month study period, with an option to remain in the study for 12 months or longer. We excluded data from participants who either withdrew consent (one participant), or completed participation without providing at least two months of data. We processed data from 130 participants, 120 of whom had provided data for at least three months, and 61 participants provided data for at least 12 months. All participants gave written informed consent to participate in the study. All patient participants were screened by an experienced psychiatrist (KEAS) using the Structured Clinical Interview for DSM IV and the borderline items of the International Personality Disorder Examination (IPDE) (Loranger et al., 1994). The study was approved by the NRES Committee East of England – Norfolk (13/EE/0288) and the Research and Development department of Oxford Health NHS Foundation Trust. The demographic details of the participants are summarised in Table 1.

We used the Wilcoxon statistical hypothesis test to assess whether there are statistically significant differences conducting pairwise comparisons between the three cohorts. We found no statistically significant differences (p>0.01) when comparing the days into the study, and the ages of the participants for the three cohorts. Similarly, there was no statistically significant difference in terms of gender between HC and BD, but gender was statistically significantly different between HC and BPD (p=0.003), and also between BD and BPD (p=0.006).

### 2.1 Established questionnaires

The participants completed the following standardized questionnaires on a *weekly* basis using the True Colours (TC) system (www.truecolours.nhs.uk) online: (i) Altman Self-rating Mania scale (ASRM) (Altman et al., 1997) to assess mania, (ii) Quick Inventory of Depressive Symptomatology Self-Report (QIDS) (Rush et al., 2003) to assess depression, (iii) Generalised Anxiety Disorder (GAD-7) (Spitzer et al., 2006) to assess anxiety, and (iv) EQ-5D (EuroQoL) assessing quality of life.

ASRM is a five-item scale requesting participants to report on (1) mood, (2) self-confidence, (3) sleep disturbance, (4) speech, and (5) activity level over the past week. Items are scored on a 0 (symptom-free) to 4 (present nearly all the time) scale, with total scores ranging from 0 to 20. Miller et al. (2009) proposed a cut-off score of 5.5 assess a manic episode on the basis that this threshold demonstrated an optimal trade-off between sensitivity and specificity.

QIDS is comprised of 16 items, which constitute nine symptom domains for depression. Each domain contributes 0–3 points, with total scores ranging from 0 to 27. The suggested clinical ranges are 5 or less denoting normal, 6–10 denoting mild depression, 11–15 denoting moderate depression, 16–20 denoting severe depression, and 21–27 denoting very severe depression.

GAD-7 contains seven items each of which is scored from 0 (symptom-free) to 3 (nearly every day), with total scores ranging from 0 to 21. Kroenke et al. (2007) endorsed using the threshold cut-offs at 5, 10, and 15 to denote mild, moderate, and severe anxiety, respectively.

EQ-5D is a standardised validated questionnaire assessing mental health status, and was developed by the EuroQol Group in order to provide a simple, generic measure of health for clinical and expedient evaluation. Only the item where participants quantify their quality of life (0–100%) was used.

### 2.2 The daily questionnaire: Mood Zoom (MZ)

**Fig. 1:**
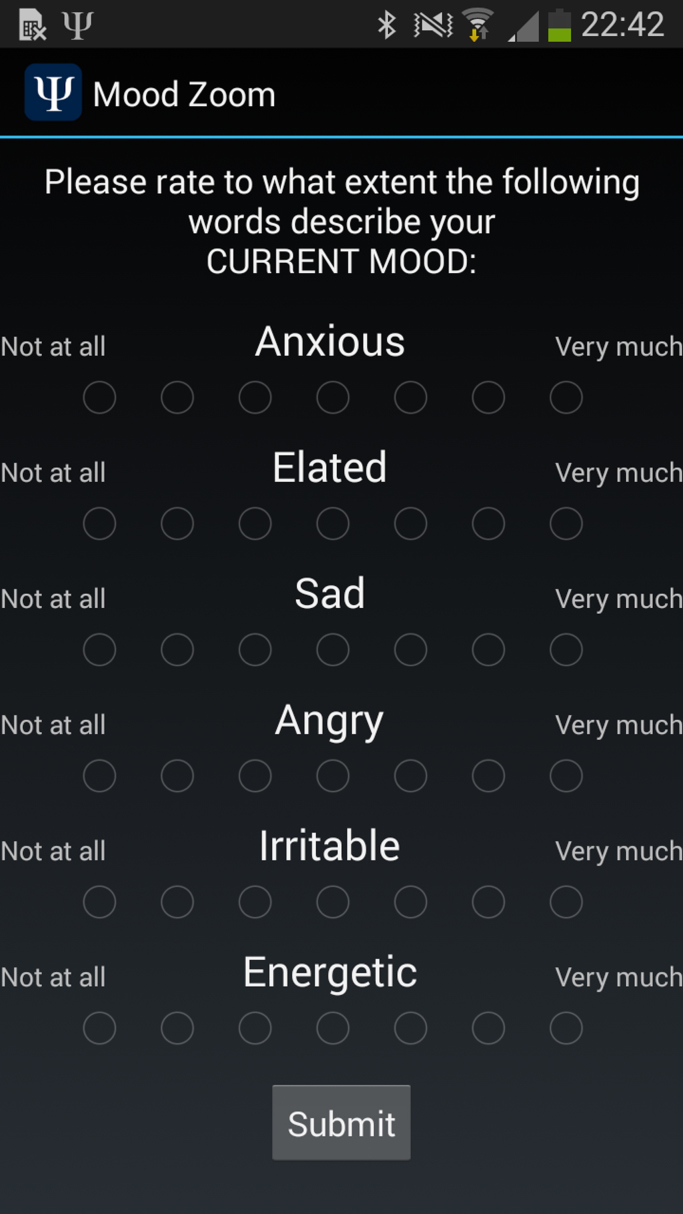
Mood Zoom questionnaire as it typically appears on a participant's phone.

MZ was conceived to identify predominant mood states, based on simple questions that can be easily answered on the smartphone's screen. It is comprised of the following six descriptor items: (1) anxious, (2) elated, (3) sad, (4) angry, (5) irritable, and (6) energetic. Participants were asked to assess the extent that each descriptor captured their mood, and to rate this on a Likert scale (1–7). The six items were based on experience sampling methodology. Participants were prompted to report their mood during the study *daily* in the evening at a pre-specified time convenient for each participant. The MZ questionnaire was implemented as part of a customised Android application developed for this study (a screenshot appears in Fig. 1).

## 3 Methods

This section summarises the main methodological attempts to understand the data.

### 3.1 Adherence

Adherence was defined as the proportion of prompted responses that were completed. For MZ it required that participants completed their daily assessment within the day of the prompt on their smartphone. For the weekly questionnaires, it required the completion of the weekly questionnaire within two days before or after the day on which we requested it be completed. In total, we processed 39,114 samples for MZ, and 7709 samples for the established weekly questionnaires.

In the Supplementary material we explore whether there is any structure in the missing entries.

### 3.2 Finding the internal structure of the new MZ questionnaire

Multidimensional data can often be described in terms of latent variable structure, for example Principal Component Analysis (PCA). Although recent research focuses on more complicated methods, PCA has the advantage that it is considerably more interpretable as a linear projection method, and also leads to a unique solution without additional fine-tuning of any hyper-parameters. It had been used previously to understand the internal structure of QIDS (Rush et al., 2006).

### 3.3 Associating MZ with the established psychiatric questionnaires

In order to validate the MZ questionnaire, we computed its statistical association against established self-report questionnaires. We used Spearman correlation coefficients to quantify the associations of each questionnaire domain for the QIDS, ASRM, GAD-7 and EQ-5D. We adopted the standard guideline in medical applications that statistical correlations with a magnitude equal or larger than 0.3 are *statistically strong* (Meyer et al., 2001, Hemphill, 2003, Tsanas et al., 2013).

The established questionnaires capture experience over the preceding week, whereas MZ is recorded on a daily basis. To allow a fair comparison we averaged the daily MZ item values over the week before the weekly ratings were made. We explored alternative approaches for summarizing the seven MZ entries and present the results in the Supplementary material.

### 3.4 Quantifying variability

Our hypothesis was that variability might discriminate BD and BPD groups from HC and more critically from each other. To quantify the *variability* of our time-series, we used the standard deviation and the Teager-Kaiser Energy Operator (TKEO), entropy, and the Root Mean Squared Successive Differences (RMSSD) for each of the six MZ items, and for each of the items of the other questionnaires. RMSSD is a fairly simple algorithmic approach to quantify variability and was recently used in a related application (Gershon and Eidelman, 2014). The TKEO has been widely used in other medical applications to identify patterns successfully (De Vos et al., 2011, Solnik et al., 2010, Tsanas, 2012). It is an application of an operator resulting in a vector output; we used the mean TKEO value to summarise its content as a scalar.

Specifically, we computed:

(1) $$\mathrm{Teager}-\mathrm{Kaiser}\;\mathrm{Energy}\;\mathrm{Operator}(\mathrm{TKEO})=\frac{1}{N}\sum_{i=2}^{N-1}\left({x_{i}^{2}-x}_{i+1}{∙x}_{i+1}\right)$$

(2) $$\mathrm{Entropy}=-\sum_{i=1}^{N}p(x_{i})∙\log(p\left(x_{i}\right))$$

(3) $$\mathrm{Root}\;\mathrm{Mean}\;\mathrm{Squared}\;\mathrm{Successive}\;\mathrm{Differences}\;(\mathrm{RMSSD})=\sqrt{\frac{1}{N}\left(\sum_{i=1}^{N-1}{\left(x_{i+1}-x_{i}\right)}^{2}\right)}$$

where N is the total number of samples for the investigated variable, and $x_{i}$ indicates a realisation of the investigated variable (e.g. QIDS or MZ).

### 3.5 Differentiating groups on the basis of TC and MZ

The questionnaires can be thought of as multivariate signals, sampled across a range of items (five items for ASRM, sixteen for QIDS, seven for GAD-7, one for EQ-5D, six for MZ). Here, we studied both the items independently, and also used the total score for each week. We studied the MZ items independently, and also using the three principal components which resulted from the application of PCA.

We computed pairwise comparisons between the three groups using the Wilcoxon rank sum statistical hypothesis test. The null hypothesis is that the samples from the two groups under investigation come from distributions with equal medians.

## 4 Results

### 4.1 Participant adherence for the weekly questionnaires and for the daily MZ questionnaire

**Fig. 2:**
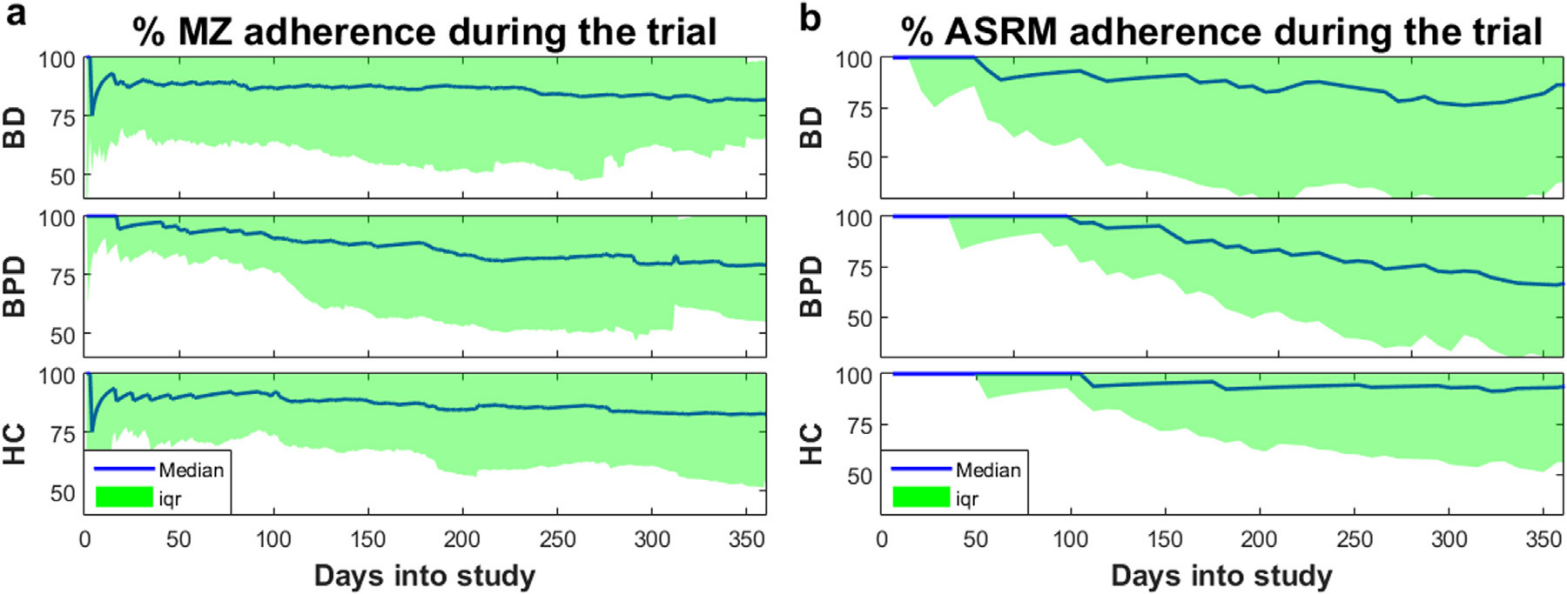
Longitudinal adherence as a function of the time into the study for each of the three groups for (a) MZ and (b) ASRM. The adherence for the other weekly questionnaires is almost identical to ASRM. We remark that participant adherence was more variable as a function of days into study, but remained very high overall even after approximately a year. The participants were originally recruited for an initial three-month study period, with an option to remain in the study for 12 months or longer; this might explain the increase in % variability beyond the first three months. However, we consider the approximately 80% adherence even at the end of the study very satisfactory.

The participant adherence throughout the study was (median±iqr%) 81.2±29.2 for MZ, and 86.3±49.8 for the weekly questionnaires. Furthermore, we tested whether participants gradually grew tired of completing the daily and weekly questionnaires. Fig. 2 presents the response rates for MZ and weekly questionnaires. Overall, these plots indicate that the overall participant adherence remained relatively stable in the study, particularly for HC and BD. Most participants completed participation in the study after one year, but some participants have provided data for considerably longer and tend to be very compliant. In order not to bias the results, we only present findings for up to one year. The adherence variability progressively increased, particularly after the third month into the study; this might reflect that participants were originally recruited for an initial three-month study period. Specifically, after the first three months MZ adherence was BD: 86.7±23.3, BPD: 92.8±15.6, HC: 92.2±17.2, and weekly questionnaire adherence was BD: 92.3±20.2, BPD: 100.0±15.2, HC: 100.00±6.58, whilst after 12 months the MZ adherence was BD: 81.9±16.7, BPD: 79.2±24.4, HC: 82.9±28.3, and weekly questionnaire adherence was BD: 86.3±49.0, BPD: 65.7±37.3, HC: 93.14±37.3.

### 4.2 Latent variable structure of the MZ questionnaire

The PCA results are summarised in Table 2, and using the first three components we can explain 85% of the variance in MZ. Moreover, the components have tentative interpretations, summarizing “negative”, “positive” and “irritability” affects in MZ. Henceforth, we denote these components as “negative MZ” (MZneg), “positive MZ” (MZpos) and “irritability MZ” (MZirr), respectively. Note that “irritability MZ” is dominated by the corresponding “irritable” and “angry” items in MZ, whilst being inversely associated with anxiety and sadness. Tables S1, S2, and S3 in the Supplementary material provide the results for the latent variable structure of MZ for each of the three groups separately; the positive and negative MZ dimensions (P1 and P2) were the same within groups (i.e. they were stable across the three cohorts) whereas P3 was less stable (the third MZ component was different for each of the three cohorts). We verified the stability of the PCA coefficients in more elaborate investigations in the Section “Investigation of the latent variable structure stability” of the Supplementary material.

### 4.3 Associating MZ with TC questionnaires

Table 3 summarises the statistical associations between the MZ items and the four established questionnaires. There are some statistically strong correlations between negative MZ items (and P1) and both items and total scores of QIDS and GAD-7, and EQ5-D. The signs of the correlations are as would be expected. Positive MZ items and P2 correlated weakly (<0.3) with ASRM items and not with other measures. P3 (‘irritability’) did not correlate well with other measures.

### 4.4 Summary statistics for the questionnaires and differences between groups

Table 4 presents summary statistics of the scores on questionnaires for the three groups for the duration of the trial. For each participant, we computed the median score for each variable, before summarizing the entries for each of the three groups. Median scores for the BD group were higher than HC for ASRM, QIDS, GAD-7 and MZneg (and lower for EQ-5D). Median scores for the BPD group were higher than HC for ASRM, QIDS, GAD-7 and MZneg (and lower for MZirr). Median scores for BPD were higher than BD for QIDS, GAD-7, MZneg and lower for MZirr and EQ-5D.

### 4.5 Variability of PROMs

Table 5 summarises the four measures of variability for the ASRM, QIDS and GAD-7 across the questionnaires during the weekly monitoring. Overall, there was much greater variation on all measures for the clinical groups compared to HC. There is some evidence for slightly greater QIDS variability in the BPD group compared to the BD group, but much greater variability in the daily measures MZneg, MZpos, MZirr in the BPD group versus BD.

## 5 Discussion

Adherence to both modalities of self-report was high (>80%) for the full observation period of 1 year. Daily MZ items of negative mood correlated highly with the scores from individual questions or total weekly scores on the QIDS and GAD-7 questionnaires. Correlations were weaker between the daily ratings of positive mood and weekly ASRM scores. Both the clinical groups (BD, BPD) exhibited greatly increased amplitudes and variability in all self-reported scores (daily and weekly) compared to HC. For weekly scores, some measures of the variability of the QIDS suggested a moderately increased effect in BPD compared to BD. For all daily scores, the BPD group showed higher variability than BD; the biggest effect was seen for variation in daily scores of irritability. Differences in variability tended to be more marked with the TKEO or RMSSD.

Our experience confirms that the intuitively appealing smartphone-based MZ questionnaire is a viable approach to be used in practice for longitudinal daily monitoring. This is in agreement with Holmes et al. (2016) who also reported that their BD cohort was very compliant in daily mood monitoring both pre- and post-treatment, although their monitoring period only lasted one month. Similarly, Schwartz et al. (2016) reported very good adherence to daily monitoring for the two-week duration of their trial. To our knowledge, this is the first time that longitudinal daily mood monitoring has been reported from such a large number of participants tracked for *many months*, as opposed to a *few weeks*. It was a community study and participants were engaging in their normal activities rather than being monitored under carefully controlled conditions, like an in-patient facility. Patients with BPD have not previously been studied in the same way. In addition to PROMs, there is an increasing body of research literature on studying mental disorders using objective behavioural and physiological signals to develop reliable biomarkers. The utility of smart phones to monitor mood in combination with the capture of phone sensor data have already revealed promising findings. Grunerbl et al. (2015) recruited ten participants who were followed for 12 weeks using smartphone-based sensor modalities. They processed social interaction, physical motion, speech, and travel pattern data to detect depressive and manic states. Mood ratings were performed by clinicians on a three weekly basis. Given the predominance of mood instability in bipolar disorder and the burden of frequent clinician assessment MZ may provide a critical link between objective sensor-derived data and mood. Similarly, there is increasing interest in the use of other sensors and additional data modalities (Lanata et al., 2015, Valenza et al., 2013).

### 5.1 The validity of MZ smart phone measures

In principle, participants could complete MZ on paper instead of a smartphone. However, there are numerous advantages to using a smartphone to collect mood data: (a) the smartphone prompts participants for completing the questionnaire, hence potentially increasing adherence, (b) the prompted response is time-stamped, so that MZ completion beyond a time window could be disregarded or processed differently, (c) alleviates the common problem of deferred completion in paper-based questionnaires, (d) the participants do not need to carry papers for mood self-assessment since everything is conveniently done on a smartphone, (e) convenience in data storage and processing, (f) the use of a smartphone opens up possibilities to record additional objective data which might be useful in mood monitoring, e.g. phone use, activity, and GPS, to complement the self-reported measures. Although it is difficult to quantify the increase in adherence as a result of using MZ as part of a smartphone application compared to a paper-based form, participant feedback suggests the reminder prompt in the MZ smartphone application increased completion rates. This is in agreement with reports in other clinical studies that adherence is improved as a result of using reminders (Fenerty et al., 2012, Vervloet et al., 2012).

The validity of the daily MZ measures of emotion was confirmed by their correlation with standard scales. There is no widely used mood monitoring questionnaire used on a daily basis against which to compare MZ. The standard mood questionnaires used in this study (ASRM, QIDS, GAD-7, EQ-5D) could have been, in principle, used on a daily basis, but they include a large number of items collectively and are comprised of lengthy questions which would be cumbersome and time-consuming to be completed daily. On the other hand, the new compact MZ questionnaire, which fits on the smartphone's screen and takes a couple of seconds to be completed, can be effectively used on a long-term daily basis. The validation of the daily MZ against the weekly questionnaires requires summarizing the seven MZ entries on a single value to be compared against each the weekly entries in the TC questionnaires. To this end, we associated the average of the seven MZ entries with TC in Table 3, and further explored alternative approaches to associate MZ and TC in the Supplementary material using: (a) the median MZ scores (S6), (b) only the last three days of MZ entries preceding the TC record (S7), and (c) the MZ entries on the same day that TC was recorded (S8). In fact, the report of weekly symptoms may be primarily related to symptoms on the day of the assessment rather than a true average of the preceding week (compare Table 3 and Table S8 in the Supplementary material). This observation, if correct, further supports the need to use higher frequency monitoring to quantify mood instability, and casts doubt on the usual assumption that weekly questionnaires encapsulates experience over the preceding week.

We also determined the latent variable MZ structure as two principal components: negative affect, and positive affect, which together accounted for almost 80% of the variance. This finding strongly confirms many studies of normal emotion in psychology, where negative and positive affects are not simply inversely proportional (Anastasi, 1982). To the best of our knowledge, this finding has not been described in such detail in BD and BPD patient groups before. In these groups, the principal component capturing negative emotion was larger than that capturing positive emotion and vice versa in HC (Supplementary Tables S1, S2, and S3), because depressed mood was more prevalent.

The negative principal component of MZ was statistically very strongly associated with depression, anxiety, and quality of life scores. All nine QIDS domains showed similar association strength (~0.4–0.7), strongly associated with the four negative MZ items (anxious, sad, angry, and irritable). In conclusion, our findings strongly support the use of MZ to capture negative mood in patients with abnormal mood and healthy volunteers.

The positive principal component of MZ was weakly correlated with scores of manic symptoms with ASRM. It is slightly surprising that ASRM items to capture happiness and activity were not more congruent with MZ scores for elation and energy. It is not obvious which is preferable. Relatively few manic symptoms (as measured by the ARSM) were reported during the study and there may be a general difficulty with capturing elated mood using self-reported assessment, as reported in previous studies (Faurholt-Jepsen et al., 2015).

We tentatively interpreted the third MZ principal component as “irritability”, but it is not stable when analysing the data within the three groups (see Tables S1, S2, S3). Nonetheless, this MZ component appears to differentiate the three groups (see Table 4); in particular BD and BPD can be distinguished better using irritability MZ than positive MZ. The results in Table 5 suggest that the first principal component of MZ is statistically very strongly associated with depression, anxiety, and quality of life scores, whilst the other principal components exhibit considerably weaker associations.

### 5.2 Differences between patients and controls

Although BD has been traditionally considered to be dominated by mania and depression, anxiety is a common comorbid factor (Simon et al., 2004). The findings in this study strongly support the argument for measuring anxiety as part of the diagnostic and monitoring protocol. The variability in the questionnaires was quantified using relatively straightforward statistical descriptors and algorithms, where TKEO and RMSSD lead to satisfactory differentiation of the three groups (see Table 5). Although it may be difficult to differentiate BD and BPD with the classical questionnaires in some cases, e.g. in terms of ASRM and GAD-7, MZ can successfully distinguish the two cohorts (see Table 5), which is of potential clinical importance (Saunders et al., 2015). This is the first study to demonstrate such a clear distinction between BD and BPD on the basis of a simple quantification of mood instability. It also highlights that different disorders require different sampling frequencies to optimally capture mood variability.

### 5.3 Overview of the different measures of variability

The variability of the longitudinal responses to the questionnaires can be quantified using time-series tools. The standard deviation is the most well-known generic descriptor to quantify variability, but may be limited in the presence of outliers or data considerably deviating from normality. The entropy is a measure of overall uncertainty, but is not sensitive to local fluctuations and relies on accurate estimation of the underlying density. RMSSD is a standard approach summarizing the successive squared differences, but only captures information contained in the amplitude changes. Finally, TKEO is a more sophisticated operator accounting for both the amplitude and the frequency of the time-series variability. These are general considerations, and in practice it is useful to apply the different operators since there is no approach which is universally best.

### 5.4 Limitations

Despite the promising findings reported in this study, there are certain limitations. Most of the BD participants were recruited from a larger study, and hence may have been more compliant than a new cohort in this diagnostic group. Nevertheless, the vast majority of participants stayed in the study beyond the originally minimum period of 3 months, which suggests that participants found the study engaging. Qualitative feedback from participants suggested that they found the completion of regular mood ratings helpful. This may have reduced the symptom burden they experienced and also have improved the recall of the weekly ratings. We also remark that while the study cohort was representative of a subgroup of psychiatric outpatients, it did not include those who were psychotic or who had significant comorbidities. This study was observational in nature and we had very little contact with participants; although we have recorded the pharmacological treatment initially, we do not have accurate information on changes in medication through the duration of the study. Given that there is emerging evidence that mood instability is associated with poor clinical outcomes in diverse mental disorders (Broome et al., 2015, Patel et al., 2015), future studies could investigate further potential differences amongst psychiatric groups. Another direction would be investigating gender- and age-based effects on mood instability, which would ideally require a larger and more balanced dataset (in particular more male BPD). Finally, all the reported scores rely on self-assessment; there is a lack of ongoing clinical assessment by experts to validate the findings: as discussed above, self-reported measures on mania may not be reflective of the true clinical condition (Faurholt-Jepsen et al., 2016).

## 6 Conclusion

The findings in this study support the use of MZ for efficient, long-term, effective daily monitoring of mood instability in clinical psychiatric practice. People diagnosed with BPD show higher ratings of distress compared to BD (or HC). The increased amplitude of ratings of negative mood and anxiety were accompanied by greater day-to-day variability in the BPD group. Such measures of mood instability may prove useful in measuring outcome in both BD and BPD patients and as a target for measuring the efficacy of drug or psychological treatment.

## Authors' contributions

(1) Conception and design of the study: AT, GDC, KEAS, GMG
(2) Writing of the clinical protocol for ethical approval: AT, KEAS
(3) Collection of data: ACB, KEAS
(4) Analysis of the data: AT
(5) Android application development: NP, MO
(6) Interpretation of findings: AT, ACB, GMG, KEAS, MDV
(7) Writing the first draft of the manuscript: AT
(8) Obtaining of funding: GDC, GMG
(9) Project oversight: GMG, GDC, MDV

All authors contributed to critically revising the manuscript for important intellectual content, and approved the final version of the manuscript to be considered for publication.

## Data access

Requests for access to the data can be made to GMG, but the data cannot be placed into a publicly accessible repository.

## Source code access

The MATLAB source code for processing the data will be made available on the first author's website. The Android application source code is available from http://amossstudy.bitbucket.org.

## Funding

The study was supported by the Wellcome Trust through a Centre Grant no. 98,461/Z/12/Z, “The University of Oxford Sleep and Circadian Neuroscience Institute (SCNi)”. This work was also funded by a Wellcome Trust Strategic Award (CONBRIO: Collaborative Oxford Network for Bipolar Research to Improve Outcomes, Reference number 102,616/Z). NP and MO acknowledge the support of the RCUK Digital Economy Programme Grant number EP/G036861/1 (Oxford Centre for Doctoral Training in Healthcare Innovation).

## Competing interests

ACB has received salaries from P1vital Ltd. GMG has held grants from Servier, received honoraria for speaking or chairing educational meetings from Abbvie, AZ, GSK, Lilly, Lundbeck, Medscape, Servier, and advised AZ, Cephalon/Teva, Lundbeck, Merck, Otsuka, P1vital, Servier, Sunovion and Takeda, holds shares in P1vital.

## Figures and Tables

**Table 1:** Summary of the AMoSS study details for the three groups.

	Bipolar Disorders (BD)	Borderline Personality Disorders (BPD)	Healthy Controls (HC)
Originally recruited	53	33	53
Processed data from	48	31	51
Days in study	353±261	313±107	276±253
Age (years)	38±21	34±15	37±20
Gender (males)	16	2	18
Any psychotropic medication	47	23	0
Lithium	19	0	0
Anticonvulsant	19	1	0
Antipsychotic	33	6	0
Antidepressants	17	23	0
Hypnotics	3	2	0

Of the 139 recruited participants, nine participants were excluded from further analysis who withdrew consent or failed to provide at least two months of data. The details provided refer to the 130 participants whose data was further processed. Where appropriate, we summarised the distributions in the form median±iqr range.

**Table 2:** Principal components to identify the latent variable structure of Mood Zoom.

	**P1**	**P2**	**P3**	**P4**	**P5**	**P6**
**Anxious**	**0.55**	0.08	**−0.47**	−0.27	0.60	0.18
**Elated**	−0.11	**0.76**	−0.11	−0.53	−0.33	0.01
**Sad**	**0.52**	0.04	**−0.43**	0.39	−0.57	−0.25
**Angry**	**0.42**	0.11	**0.46**	0.11	−0.21	0.74
**Irritable**	**0.47**	0.12	**0.60**	−0.15	0.14	−0.60
**Energetic**	−0.13	**0.62**	0.02	0.67	0.38	−0.03
**% Total variance explained**	55	77	85	91	97	100
**Tentative interpretation**	“Negative feelings”	“Positive feelings”	“Irritability”			

Bold entries indicate the loadings which dominate each principal component.

**Table 3:** Statistical associations (Spearman correlation coefficient) between MZ and the constituent items and total scores of the established weekly questionnaires (ASRM, QIDS, GAD-7, EQ-5D).

		**MZ items**						**MZ factors**		
		**Anxious**	**Elated**	**Sad**	**Angry**	**Irritable**	**Energetic**	**Negative**	**Positive**	**Irritability**
**ASRM**	**Happy**	0.08	0.26	0.07	0.07	0.11	0.19	0.06	0.26	−0.06
	**Confident**	0.09	0.26	0.05	0.08	0.12	0.19	0.06	0.26	−0.04
	**Sleep**	0.18	0.16	0.15	0.18	0.24	0.08	0.18	0.17	−0.01
	**Talkative**	0.16	0.21	0.14	0.15	0.20	0.11	0.16	0.21	−0.06
	**Active**	0.14	0.21	0.12	0.13	0.15	0.19	0.12	0.24	−0.06
**QIDS**	**Sleep**	**0.38**	−0.08	**0.33**	**0.31**	**0.34**	−0.13	**0.39**	0	−0.02
	**Sad**	**0.65**	−0.01	**0.76**	**0.55**	**0.53**	−0.16	**0.71**	0.08	**−0.30**
	**Appetite/weight**	**0.46**	−0.02	**0.39**	**0.35**	**0.39**	−0.17	**0.46**	0.02	−0.09
	**Concentration**	**0.59**	−0.09	**0.56**	**0.46**	**0.49**	−0.23	**0.61**	−0.02	−0.18
	**Self-view**	**0.59**	−0.03	**0.63**	**0.45**	**0.46**	−0.18	**0.62**	0.04	−0.25
	**Suicide**	**0.47**	−0.06	**0.56**	**0.41**	**0.39**	−0.17	**0.53**	0.01	−0.16
	**Interest**	**0.52**	−0.07	**0.57**	**0.41**	**0.43**	−0.20	**0.56**	0	−0.18
	**Energy**	**0.54**	−0.12	**0.55**	**0.39**	**0.42**	−0.27	**0.57**	−0.06	−0.21
	**Restless**	**0.57**	−0.04	**0.55**	**0.44**	**0.49**	−0.15	**0.60**	0.05	−0.15
**GAD-7**	**Nervous/anxious**	**0.72**	0	**0.64**	**0.53**	**0.55**	−0.16	**0.69**	0.08	−0.25
	**Control worries**	**0.67**	0	**0.66**	**0.54**	**0.53**	−0.14	**0.67**	0.1	−0.24
	**Worried**	**0.69**	0.01	**0.66**	**0.54**	**0.54**	−0.13	**0.67**	0.11	−0.25
	**Relaxed**	**0.68**	−0.02	**0.62**	**0.51**	**0.55**	−0.15	**0.67**	0.08	−0.22
	**Restless**	**0.54**	0.09	**0.50**	**0.44**	**0.45**	−0.04	**0.54**	0.16	−0.15
	**Irritable**	**0.63**	0.07	**0.58**	**0.61**	**0.69**	−0.12	**0.67**	0.16	0
	**Afraid**	**0.67**	−0.04	**0.67**	**0.54**	**0.54**	−0.17	**0.68**	0.07	−0.2
**EQ-5D**		**−0.58**	0.15	**−0.55**	**−0.46**	**−0.50**	0.37	**−0.63**	0.11	0.09
**Total**	**ASRM**	0.19	0.26	0.16	0.17	0.22	0.17	0.17	0.27	−0.07
	**QIDS**	**0.67**	−0.05	**0.69**	**0.53**	**0.56**	−0.22	**0.71**	0.03	−0.23
	**GAD-7**	**0.77**	0.03	**0.72**	**0.61**	**0.65**	−0.15	**0.77**	0.13	−0.23

Bold entries indicate statistically strong associations (Spearman |R|≥0.3). All entries with |R|≥0.1 were statistically significant (p<0.0001). We used the nine QIDS domains rather than the 16 items, because depression is clinically assessed in this way. Each of the items of the weekly questionnaires is presented as a sentence to participants; we present these as words here to facilitate comparisons. The MZ factors were determined using the PCA loadings computed in Table 2.

**Table 4:** Summary statistics of the questionnaires used in the study, and statistical significance pairwise comparisons across the three groups (BD, BPD, HC) using the Wilcoxon statistical hypothesis test.

		**BD** (median±iqr)	**BPD** (median±iqr)	**HC** (median±iqr)	**BD vs BPD** (*p*-value)	**BD vs HC** (*p*-value)	**BPD vs HC** (*p*-value)
**Total**	**ASRM**	1.00±3.00	1.00±2.00	0.00±1.00	0.8128	**0.0008**	**0.0023**
	**QIDS**	6.25±6.75	14.50±5.88	1.00±2.25	**6.0194e−08**	**2.9603e−12**	**1.1331e−13**
	**GAD-7**	5.00±6.00	12.00±9.00	0.00±1.00	**1.1245e−05**	**1.1962e−10**	**2.8181e−14**
	**EQ-5D**	68.00±18.75	60.00±21.50	85.00±16.00	**0.0225**	**2.6322e−08**	**4.6093e−11**
**MZ**	**MZneg**	3.58±2.31	6.44±3.27	1.73±1.39	**5.4323e−05**	**3.4962e−06**	**4.1247e−11**
	**MZpos**	4.05±1.92	4.85±2.26	4.20±2.67	0.2608	0.9163	0.3973
	**MZirr**	−0.16±0.71	−0.47±1.41	0.00±0.42	**0.0116**	0.2949	**0.0032**

**Table 5:** Comparing variability during the low monitoring period across the three groups, and statistical significance pairwise comparisons across the three groups (BD, BPD, HC) using the Wilcoxon statistical hypothesis test.

	**BD** (median±iqr)	**BPD** (median±iqr)	**HC** (median±iqr)	**BD vs BPD** (*p*-value)	**BD vs HC** (*p*-value)	**BPD vs HC** (*p*-value)
**ASRM**_**std**_	2.40±1.99	2.03±1.55	0.87±1.36	0.7291	**1.0506e−07**	**1.7383e−06**
**ASRM**_**TKEO**_	2.73±6.46	3.87±7.34	0.78±2.09	0.4646	**8.4909e−05**	**3.6978e−06**
**ASRM**_**RMSSD**_	2.47±1.00	2.32±0.48	1.84±0.82	0.4424	**1.1898e−05**	**0.0001**
**ASRM**_**entropy**_	1.81±1.26	2.33±1.71	0.91±1.43	0.3204	**8.4268e−07**	**3.8933e−07**
**QIDS**_**std**_	3.39±2.29	3.54±1.67	1.15±0.91	0.1834	**2.7408e−10**	**3.5538e−11**
**QIDS**_**TKEO**_	10.45±16.87	18.64±21.05	1.14±3.57	**0.0080**	**7.1641e−07**	**5.6639e−10**
**QIDS**_**RMSSD**_	2.83±2.06	3.63±1.63	1.37±0.93	**0.0161**	**5.1287e−08**	**3.8656e−10**
**QIDS**_**entropy**_	2.61±0.73	2.81±0.60	1.94±0.64	**0.0400**	**7.4278e−07**	**6.7001e−09**
**GAD-7**_**std**_	3.17±2.06	2.86±1.47	0.88±0.96	0.2701	**4.7697e−12**	**7.6226e−09**
**GAD-7**_**TKEO**_	7.71±11.88	12.45±14.75	0.85±2.77	0.2667	**7.4507e−09**	**2.1025e−07**
**GAD-7**_**RMSSD**_	2.73±1.61	3.11±1.22	1.03±0.96	0.9292	**1.0668e−10**	**3.8804e−08**
**GAD-7**_**entropy**_	2.66±0.68	2.53±0.46	1.76±0.85	0.1089	**2.3571e−10**	**4.9003e−07**
**EQ5D**_**std**_	9.48±9.70	11.74±7.60	5.04±4.42	0.2746	**1.7334e−06**	**6.9127e−08**
**EQ5D**_**TKEO**_	290.17±425.40	283.13±329.10	388.04±554.98	0.6457	**0.0357**	**0.008**
**EQ5D**_**RMSSD**_	8.55±9.97	11.78±9.25	5.40±4.53	**0.0282**	**5.3213e−05**	**3.7823e−08**
**EQ5D**_**entropy**_	3.68±0.76	3.91±0.63	3.20±0.83	0.1206	**7.4667e−05**	**3.2071e−06**
**MZneg**_**std**_	1.83±1.02	2.13±0.94	0.80±0.92	**0.0166**	**3.8065e−08**	**1.9054e−11**
**MZneg**_**TKEO**_	2.02±2.03	4.04±2.86	0.47±0.97	**0.0002**	**1.4284e−07**	**2.0344e−11**
**MZneg**_**RMSSD**_	1.77±0.86	2.37±0.69	0.86±0.89	**0.0001**	**6.0851e−07**	**1.5645e−11**
**MZneg**_**entropy**_	1.94±0.66	2.21±0.40	1.16±1.25	**0.0040**	**1.6629e−07**	**3.2102e−11**
**MZpos**_**std**_	1.33±0.55	1.53±0.73	0.88±0.49	0.0747	**0.0002**	**1.2968e−05**
**MZpos**_**TKEO**_	1.25±1.24	1.82±1.93	0.73±0.96	**0.0329**	**0.0055**	**6.6719e−05**
**MZpos**_**RMSSD**_	1.38±0.69	1.69±0.85	0.89±0.56	**0.0041**	**0.0015**	**2.3122e−06**
**MZpos**_**entropy**_	1.73±0.47	1.84±0.54	1.41±0.61	**0.0451**	**0.0227**	**0.00051794**
**MZirr**_**std**_	0.98±0.36	1.19±0.41	0.48±0.46	**4.6748e−05**	**8.6713e−08**	**5.0433e−11**
**MZirr**_**TKEO**_	0.66±0.57	1.09±1.02	0.20±0.36	**7.7156e−06**	**6.5418e−07**	**1.5792e−10**
**MZirr**_**RMSSD**_	1.09±0.46	1.39±0.57	0.61±0.54	**1.2282e−05**	**6.3094e−07**	**2.1572e−10**
**MZirr**_**entropy**_	1.35±0.46	1.60±0.37	0.86±1.12	**0.0001**	**1.9641e−05**	**1.3647e−08**

Statistically significant differences at the *p*=0.05 level appear in bold. “MZneg” denotes the negative factor of MZ, “MZpos” denotes the positive factor of MZ, and “MZirr” the irritability factor of MZ computed using the PCA loadings (see Table 2).

## Footnotes

Appendix A Supplementary data associated with this article can be found in the online version at 10.1016/j.jad.2016.06.065.
