# Supplementary material for: Daily longitudinal self-monitoring of mood variability in bipolar disorder and borderline personality disorder
Source: J Affect Disord. 2016 Nov 15;205:225–33. doi: 10.1016/j.jad.2016.06.065 (PMC5296237; doi:10.1016/j.jad.2016.06.065)
Supplement: Supplementary file 1 — Supplementary material [file mmc1.docx]

Supplementary Material

Daily longitudinal self-monitoring of mood variability in bipolar disorder and borderline personality disorder

A. Tsanas^1,2,3,*^, K.E.A. Saunders^4^, A.C. Bilderbeck^4^, N. Palmius^1^, M. Osipov, G.D. Clifford^5,6^, G.Μ. Goodwin^4^, M. De Vos^1,3^

^1^Institute of Biomedical Engineering, Department of Engineering Science, University of Oxford, UK, ^2^Oxford Centre for Industrial and Applied Mathematics, Mathematical Institute, University of Oxford, UK, ^3^Sleep and Circadian Neuroscience Institute, Nuffield Department of Clinical Neurosciences, ^4^Department of Psychiatry, University of Oxford, UK, ^5^Department of Biomedical Informatics, Emory University, Atlanta, Georgia, USA, ^6^Department of Biomedical Engineering, Georgia Institute of Technology, USA

**Keywords:** Bipolar disorder, borderline personality disorder, depression, digital health, mania, mood assessment, mood monitoring, patient reported outcome measures.

*Author for correspondence ([athanasios.tsanas@eng.ox.ac.uk](mailto:athanasios.tsanas@eng.ox.ac.uk), [tsanas@maths.ox.ac.uk](mailto:tsanas@maths.ox.ac.uk), [tsanasthanasis@gmail.com](mailto:tsanasthanasis@gmail.com)).

Present address: Institute of Biomedical Engineering, Department of Engineering Science, University of Oxford, Old Road Campus Research Building,, Headington, Oxford, UK, OX3 7DQ

Missing values

In general, missing entries in clinical settings may contain clinically useful information, but it is usually difficult to determine how this could be further explored. Here, it would be reasonable to hypothesize that participants might miss MZ assessments when their symptoms fluctuate wildly or when there is a positive/negative trend beyond some threshold, that is, missing MZ might be a function of mood. Hence, we assessed whether there is any systematic difference between MZ entries recorded immediately before and immediately after missing entries (see Figure S1). For simplicity, the presented results are for the case where the participants missed only one consecutive MZ recording. However, the results are practically identical for the cases where they have missed multiple consecutive MZ self-monitoring assessments. To get a better idea whether such differences are directly related to negative or positive MZ affects, we present the two MZ factors for each of the subjects in the study. Visually, these findings suggest there is no clear tendency that participants do not complete MZ on the basis of mood. For example, computing the average difference in the negative MZ with respect to zero leads to a value which is practically zero indicating that there is no clear trend in increasing or decreasing negative MZ as a result of which the participant has failed to complete a daily reporting. Plotting the distribution for the differences results in a nice bell-shaped curve centered on zero with small bandwidth (results not shown). This finding confirms statistically that the MZ differences are not indicative of an increasing or decreasing trend. Similar findings were observed for the positive MZ.

Overall, for most participants the average successive MZ differences are around zero, and the variance is neither positively nor negatively skewed for either MZ factor. This may be a compelling finding, which could indicate some plausible solutions towards imputing missing values, e.g. with a linearly interpolated MZ score if required. In this study, this was not explored further.

**
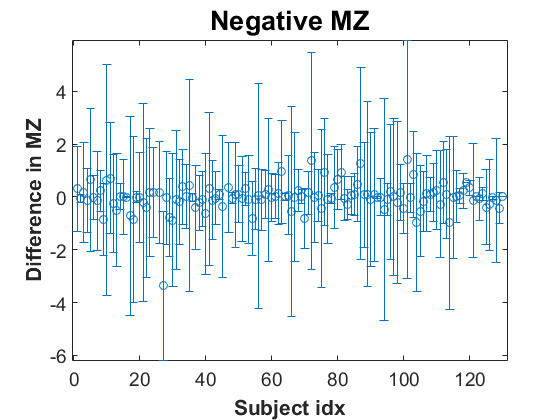

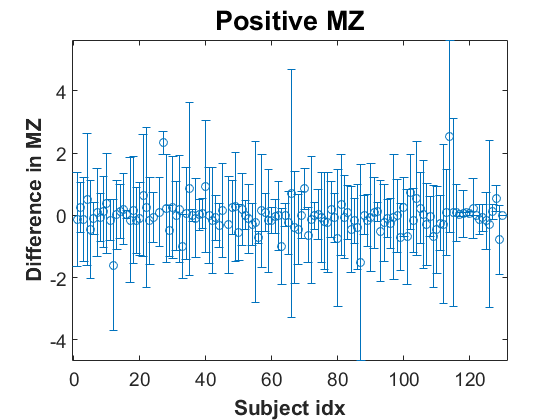
**

**Figure S1**: Successive differences in MZ factors before and after missing entries for negative MZ and positive MZ. The circles denote the mean, and the bars denote the standard deviation.

Latent variable structure of the MZ questionnaire for each of the three groups

In this section we repeat the computation of the latent variable MZ structure as in the main manuscript. Instead of providing the results for all the participants in the study, here we study independently the three cohorts (BD, BPD, HC) to assess whether there are differences in the principal components for the groups, particularly to compare whether there are differences between the clinical groups, and between the clinical groups and HC.

**Table S1**: Principal components to identify the latent variable structure of MZ in BD.

|  | **P1** | **P2** | **P3** | **P4** | **P5** | **P6** |
| --- | --- | --- | --- | --- | --- | --- |
| **Anxious** | **0.51** | 0.10 | **0.82** | -0.16 | 0.15 | 0.12 |
| **Elated** | -0.18 | **0.73** | 0.08 | -0.28 | **-0.59** | 0.02 |
| **Sad** | **0.49** | 0.07 | -0.06 | **0.70** | **-0.41** | **-0.31** |
| **Angry** | **0.46** | 0.15 | **-0.43** | -0.02 | 0.02 | 0.77 |
| **Irritable** | **0.47** | 0.19 | **-0.38** | **-0.48** | 0.25 | -**0.55** |
| **Energetic** | -0.18 | **0.63** | 0.00 | **0.42** | **0.63** | -0.02 |
| **% variance explained** | 58 | 78 | 85 | 91 | 97 | 100 |
| **Tentative interpretation** | “Negative feelings” | “Positive feelings” | “Anxiety” |  |  |  |

In bold we have highlighted the loadings which dominate each principal component.

**Table S2**: Principal components to identify the latent variable structure of MZ in BPD.

|  | **P1** | **P2** | **P3** | **P4** | **P5** | **P6** |
| --- | --- | --- | --- | --- | --- | --- |
| **Anxious** | **0.53** | -0.04 | **0.39** | **0.72** | -0.05 | 0.20 |
| **Elated** | -0.11 | **0.70** | 0.27 | -0.07 | **-0.65** | -0.04 |
| **Sad** | **0.48** | -0.24 | **0.55** | **-0.60** | -0.03 | -0.22 |
| **Angry** | **0.46** | 0.24 | **-0.37** | **-0.32** | 0.01 | **0.70** |
| **Irritable** | **0.51** | 0.26 | **-0.50** | 0.09 | 0.01 | **-0.65** |
| **Energetic** | -0.05 | **0.57** | **0.30** | -0.03 | **0.76** | -0.03 |
| **% variance explained** | 50 | 71 | 82 | 90 | 96 | 100 |
| **Tentative interpretation** | “Negative feelings” | “Positive feelings” | “Sadness” |  |  |  |

In bold we have highlighted the loadings which dominate each principal component.

**Table S3**: Principal components to identify the latent variable structure of MZ in HC.

|  | **P1** | **P2** | **P3** | **P4** | **P5** | **P6** |
| --- | --- | --- | --- | --- | --- | --- |
| **Anxious** | 0.12 | **0.56** | -0.03 | **0.72** | -0.36 | 0.13 |
| **Elated** | **0.78** | -0.11 | **-0.61** | -0.08 | -0.01 | -0.00 |
| **Sad** | 0.12 | **0.51** | 0.04 | 0.03 | **0.78** | **-0.34** |
| **Angry** | 0.10 | **0.39** | 0.10 | **-0.40** | 0.10 | **0.81** |
| **Irritable** | 0.07 | **0.47** | 0.09 | **-0.55** | **-0.51** | **-0.46** |
| **Energetic** | **0.59** | -0.20 | **0.78** | 0.09 | -0.02 | -0.03 |
| **% variance explained** | 52 | 77 | 87 | 93 | 98 | 100 |
| **Tentative interpretation** | “Positive feelings” | “Negative feelings” | “Energetic” |  |  |  |

In bold we have highlighted the loadings which dominate each principal component.

Collectively, the results in Tables S1, S2, and S3 suggest that the first two principal components are very stable across the three groups (and also with respect to the principal components reported in Table 2 in the manuscript). Crucially, the loadings are in excellent agreement for both the negative MZ and positive MZ across the three groups. For the clinical cohorts (BD, BPD) the “negative MZ” is the first principal component, whereas for the HC the “positive MZ” is the first principal component. This suggests that most of the variance in the MZ for HC is due to the positive MZ items (“elated” and “energetic”), whereas for the two clinical groups most of the variance in the data is explained collectively by the negatively-associated items (“anxious”, “sad”, “angry”, and “irritable”). Overall, jointly the two components are very similar and explain over 70% of the variance. For HC and BD they explain 77% and 78% of the variance, respectively, whereas for the BPD group they explain 71%, which may indicate that the BPD group is more variable. This hypothesis is further corroborated by the fact that the third principal component in Table S2 is a function of five items, whereas the third principal component is dominated by three items in BD (see Table S1) and only two items in HC (Table S3).

The third principal component derived is considerably more variable amongst the three groups, which complicates interpretation (see also Table 2 in the manuscript). Therefore, although we can be reasonably confident in the first two principal components of MZ identified using PCA, the use of the third principal component is considerably more tenuous.

Investigation of the latent variable structure stability

In this section we investigate the stability of the PCA findings reported in Table 2 in the manuscript. Specifically, we wanted to investigate the stability of the coefficients when using sub-sampled versions of the data. We followed two strategies: (a) randomly removing 50% of the original samples, and (b) randomly removing the samples from 30 of the 130 subjects. In each case, we repeated the process 100 times for statistical confidence, and report the findings in the form median±iqr for each PCA coefficient across the 100 repetitions. We note that the low iqr values suggest that the coefficients reported in Table 2 capture very well the latent variable structure and may generalize well in new, unseen data.

**Table S4**: Principal components to identify the latent variable structure of Mood Zoom by randomly selecting 50% of the original samples.

|  | **P1** | **P2** | **P3** | **P4** | **P5** | **P6** |
| --- | --- | --- | --- | --- | --- | --- |
| **Anxious** | **0.55±0.00** | 0.08±0.01 | **-0.47±0.02** | -0.47±0.11 | -0.47±0.07 | -0.47±0.01 |
| **Elated** | -0.11±0.01 | **0.76±0.00** | -0.11±0.02 | -0.11±0.06 | -0.11±0.10 | -0.11±0.01 |
| **Sad** | **0.52±0.00** | 0.04±0.01 | **-0.44±0.02** | -0.44±0.11 | -0.44±0.07 | -0.44±0.01 |
| **Angry** | **0.42±0.00** | 0.11±0.00 | **0.46±0.01** | 0.46±0.04 | 0.46±0.03 | 0.46±0.01 |
| **Irritable** | **0.47±0.00** | 0.12±0.01 | **0.60±0.01** | 0.60±0.03 | 0.60±0.03 | 0.60±0.01 |
| **Energetic** | -0.13±0.00 | **0.62±0.00** | 0.02±0.03 | 0.02±0.07 | 0.02±0.13 | 0.02±0.01 |
| **% Total variance explained** | 54.69±0.38 | 77.47±0.23 | 85.28±0.18 | 91.27±0.09 | 96.95±0.05 | 100.00±0.00 |
| **Tentative interpretation** | “Negative feelings” | “Positive feelings” | “Irritability” |  |  |  |

Bold entries indicate the loadings which dominate each principal component. The random sampling from the original dataset was repeated 100 times for statistical confidence, and the results from the 100 repetitions are summarized in the format median±iqr. The results are almost identical to Table 2 in the manuscript.

**Table S5**: Principal components to identify the latent variable structure of Mood Zoom by randomly removing 30 participants.

|  | **P1** | **P2** | **P3** | **P4** | **P5** | **P6** |
| --- | --- | --- | --- | --- | --- | --- |
| **Anxious** | **0.55±0.02** | 0.08±0.03 | **-0.44±0.07** | -0.44±0.37 | -0.44±0.91 | -0.44±0.03 |
| **Elated** | -0.11±0.03 | **0.76±0.02** | -0.12±0.05 | -0.12±0.22 | -0.12±0.39 | -0.12±0.02 |
| **Sad** | **0.52±0.01** | 0.03±0.03 | **-0.44±0.08** | -0.44±0.35 | -0.44±0.96 | -0.44±0.04 |
| **Angry** | **0.42±0.02** | 0.11±0.03 | **0.45±0.03** | 0.45±0.15 | 0.45±0.32 | 0.45±0.02 |
| **Irritable** | **0.47±0.01** | 0.12±0.03 | **0.60±0.04** | 0.60±0.09 | 0.60±0.24 | 0.60±0.02 |
| **Energetic** | -0.13±0.03 | **0.62±0.02** | 0.03±0.07 | 0.03±0.26 | 0.03±0.47 | 0.03±0.02 |
| **% Total variance explained** | 55.16±3.31 | 77.68±1.60 | 85.44±1.01 | 91.47±0.57 | 97.00±0.24 | 100.00±0.00 |
| **Tentative interpretation** | “Negative feelings” | “Positive feelings” | “Irritability” |  |  |  |

Bold entries indicate the loadings which dominate each principal component. The random removal of participants from the original dataset was repeated 100 times for statistical confidence, and the results from the 100 repetitions are summarized in the format median±iqr. The results are almost identical to Table 2 in the manuscript.

Associating MZ with the established psychiatric questionnaires

As discussed in the manuscript, the established questionnaires (ASRM, QIDS, GAD-7) are typically recorded every week, whereas MZ is recorded on a daily basis, which complicates direct comparison. In Table 3 we averaged the MZ entries for every week in order to obtain a single entry and associate it with the established questionnaires. Here, we explored three additional, different strategies to summarize the daily MZ entries on a single entry to compare against each weekly recorded score for the established questionnaires: (a) summarizing MZ on the basis of median values for each of the MZ items (Table S6), (b) averaging MZ from the last three days preceding the TC record (Table S7), (c) using the MZ entries on the same day that TC was recorded (Table S8). The results are quite similar in all cases.

**Table S6**: Statistical associations (Spearman correlation coefficient) between MZ and the constituent items of the established weekly questionnaires (ASRM, QIDS, GAD-7, EQ-5D), by summarizing the daily MZ using the median operator to match the weekly scores.

|  | | MZ items | | | | | | MZ factors | | |
| --- | --- | --- | --- | --- | --- | --- | --- | --- | --- | --- |
|  |  | Anxious | Elated | Sad | Angry | Irritable | Energetic | negative | positive | irritability |
| ASRM | Happy | 0.06 | 0.27 | 0.03 | 0.03 | 0.07 | 0.20 | 0.02 | 0.27 | -0.06 |
|  | Confident | 0.07 | 0.26 | 0.02 | 0.05 | 0.08 | 0.20 | 0.03 | 0.27 | -0.03 |
|  | Sleep | 0.15 | 0.13 | 0.13 | 0.16 | 0.21 | 0.09 | 0.17 | 0.17 | 0.01 |
|  | Talkative | 0.15 | 0.20 | 0.11 | 0.11 | 0.15 | 0.12 | 0.13 | 0.21 | -0.05 |
|  | Active | 0.13 | 0.20 | 0.10 | 0.09 | 0.11 | 0.22 | 0.10 | 0.26 | -0.08 |
| QIDS | Sleep | **0.35** | -0.10 | **0.30** | 0.26 | **0.31** | -0.12 | **0.36** | -0.01 | -0.02 |
|  | Sad | **0.61** | -0.08 | **0.75** | **0.49** | **0.49** | -0.18 | **0.70** | 0.04 | -0.29 |
|  | Appetite/weight | **0.44** | -0.06 | **0.38** | **0.31** | **0.36** | -0.17 | **0.45** | 0 | -0.11 |
|  | Concentration | **0.57** | -0.14 | **0.53** | **0.40** | **0.46** | -0.24 | **0.60** | -0.05 | -0.17 |
|  | Self-view | **0.56** | -0.08 | **0.63** | **0.42** | **0.43** | -0.19 | **0.62** | 0.02 | -0.25 |
|  | Suicide | **0.44** | -0.11 | **0.56** | **0.40** | **0.37** | -0.18 | **0.52** | -0.02 | -0.15 |
|  | Interest | **0.48** | -0.12 | **0.55** | **0.37** | **0.40** | -0.21 | **0.55** | -0.03 | -0.19 |
|  | Energy | **0.52** | -0.15 | **0.54** | **0.36** | **0.39** | -0.27 | **0.56** | -0.08 | -0.21 |
|  | Restless | **0.56** | -0.09 | **0.54** | **0.41** | **0.47** | -0.15 | **0.59** | 0.03 | -0.16 |
| GAD-7 | Nervous/anxious | **0.71** | -0.07 | **0.62** | **0.48** | **0.53** | -0.17 | **0.70** | 0.05 | -0.25 |
|  | Control worries | **0.66** | -0.07 | **0.65** | **0.50** | **0.52** | -0.15 | **0.67** | 0.07 | -0.24 |
|  | Worried | **0.66** | -0.05 | **0.65** | **0.49** | **0.51** | -0.14 | **0.67** | 0.08 | -0.25 |
|  | Relaxed | **0.66** | -0.08 | **0.62** | **0.48** | **0.53** | -0.16 | **0.68** | 0.06 | -0.22 |
|  | Restless | **0.53** | 0.03 | **0.50** | **0.42** | **0.44** | -0.06 | **0.54** | 0.13 | -0.15 |
|  | Irritable | **0.60** | 0.01 | **0.55** | **0.55** | **0.69** | -0.13 | **0.67** | 0.13 | 0.01 |
|  | Afraid | **0.65** | -0.10 | **0.67** | **0.51** | **0.51** | -0.18 | **0.68** | 0.04 | -0.21 |
| EQ-5D | | **-0.56** | 0.20 | **-0.54** | **-0.41** | **-0.48** | **0.37** | **-0.62** | 0.14 | 0.10 |
| Total | ASRM | 0.17 | 0.25 | 0.12 | 0.12 | 0.17 | 0.19 | 0.14 | 0.28 | -0.07 |
|  | QIDS | **0.63** | -0.11 | **0.66** | **0.46** | **0.52** | -0.22 | **0.69** | 0 | -0.23 |
|  | GAD-7 | **0.74** | -0.05 | **0.69** | **0.55** | **0.62** | -0.16 | **0.77** | 0.09 | -0.23 |

Bold entries indicate statistically strong associations (Spearman $\left| R \right|\geq0.3$). We used the nine QIDS domains rather than the 16 items, because depression is clinically assessed in this way. Each of the items of the weekly questionnaires is presented as a sentence to participants; we present these as words here to facilitate comparisons. The MZ factors were determined using the PCA loadings computed in Table 2 in the manuscript.

**Table S7**: Statistical associations (Spearman correlation coefficient) between MZ and the constituent items of the established weekly questionnaires (ASRM, QIDS, GAD-7, EQ-5D), by summarizing the daily MZ using the average of the three days closest to the TC entry to match the weekly scores.

|  | | MZ items | | | | | | MZ factors | | |
| --- | --- | --- | --- | --- | --- | --- | --- | --- | --- | --- |
|  |  | Anxious | Elated | Sad | Angry | Irritable | Energetic | negative | positive | irritability |
| ASRM | Happy | 0.06 | 0.28 | 0.02 | 0.04 | 0.08 | 0.21 | 0.02 | 0.28 | -0.06 |
|  | Confident | 0.08 | 0.27 | 0.01 | 0.05 | 0.09 | 0.21 | 0.03 | 0.28 | -0.04 |
|  | Sleep | 0.16 | 0.14 | 0.13 | 0.16 | 0.22 | 0.09 | 0.17 | 0.17 | -0.01 |
|  | Talkative | 0.15 | 0.21 | 0.11 | 0.12 | 0.18 | 0.13 | 0.13 | 0.22 | -0.05 |
|  | Active | 0.13 | 0.22 | 0.09 | 0.1 | 0.11 | 0.23 | 0.09 | 0.27 | -0.08 |
| QIDS | Sleep | **0.35** | -0.1 | **0.32** | 0.28 | **0.32** | -0.14 | **0.37** | -0.03 | -0.02 |
|  | Sad | **0.62** | -0.06 | **0.76** | **0.52** | **0.52** | -0.18 | **0.71** | 0.04 | -0.27 |
|  | Appetite/weight | **0.45** | -0.06 | **0.38** | **0.32** | **0.36** | -0.17 | **0.45** | 0 | -0.11 |
|  | Concentration | **0.57** | -0.12 | **0.53** | **0.43** | **0.47** | -0.24 | **0.60** | -0.05 | -0.15 |
|  | Self-view | **0.57** | -0.07 | **0.63** | **0.44** | **0.45** | -0.19 | **0.62** | 0.01 | -0.23 |
|  | Suicide | **0.45** | -0.11 | **0.56** | **0.41** | **0.38** | -0.18 | **0.53** | -0.02 | -0.14 |
|  | Interest | **0.49** | -0.11 | **0.56** | **0.39** | **0.41** | -0.21 | **0.55** | -0.03 | -0.17 |
|  | Energy | **0.52** | -0.14 | **0.54** | **0.37** | **0.41** | -0.28 | **0.56** | -0.08 | -0.19 |
|  | Restless | **0.56** | -0.08 | **0.54** | **0.42** | **0.47** | -0.16 | **0.59** | 0.02 | -0.15 |
| GAD-7 | Nervous/anxious | **0.70** | -0.05 | **0.63** | **0.50** | **0.53** | -0.17 | **0.69** | 0.05 | -0.23 |
|  | Control worries | **0.67** | -0.05 | **0.65** | **0.51** | **0.52** | -0.15 | **0.67** | 0.07 | -0.22 |
|  | Worried | **0.67** | -0.03 | **0.65** | **0.51** | **0.52** | -0.14 | **0.67** | 0.09 | -0.23 |
|  | Relaxed | **0.66** | -0.06 | **0.62** | **0.50** | **0.54** | -0.16 | **0.68** | 0.06 | -0.21 |
|  | Restless | **0.53** | 0.06 | **0.49** | **0.42** | **0.44** | -0.05 | **0.53** | 0.15 | -0.14 |
|  | Irritable | **0.60** | 0.03 | **0.58** | **0.60** | **0.70** | -0.14 | **0.68** | 0.13 | 0.03 |
|  | Afraid | **0.64** | -0.08 | **0.66** | **0.53** | **0.52** | -0.18 | **0.68** | 0.04 | -0.18 |
| EQ-5D | | **-0.57** | 0.19 | **-0.55** | **-0.43** | **-0.49** | **0.38** | **-0.63** | 0.14 | 0.10 |
| Total | ASRM | 0.17 | 0.27 | 0.12 | 0.14 | 0.18 | 0.21 | 0.13 | **0.30** | -0.08 |
|  | QIDS | **0.64** | -0.10 | **0.67** | **0.50** | **0.54** | -0.23 | **0.70** | -0.01 | -0.21 |
|  | GAD-7 | **0.75** | -0.02 | **0.71** | **0.58** | **0.63** | -0.17 | **0.76** | 0.1 | -0.21 |

Bold entries indicate statistically strong associations (Spearman $\left| R \right|\geq0.3$). We used the nine QIDS domains rather than the 16 items, because depression is clinically assessed in this way. Each of the items of the weekly questionnaires is presented as a sentence to participants; we present these as words here to facilitate comparisons. The MZ factors were determined using the PCA loadings computed in Table 2 in the manuscript.

**Table S8**: Statistical associations (Spearman correlation coefficient) between MZ and the constituent items of the established weekly questionnaires (ASRM, QIDS, GAD-7, EQ-5D), using only MZ assessments on the same day of the TC entry to match the weekly scores.

|  | | MZ items | | | | | | MZ factors | | |
| --- | --- | --- | --- | --- | --- | --- | --- | --- | --- | --- |
|  |  | Anxious | Elated | Sad | Angry | Irritable | Energetic | negative | positive | irritability |
| ASRM | Happy | 0.04 | **0.30** | 0.01 | 0.01 | 0.06 | 0.21 | -0.01 | 0.29 | -0.05 |
|  | Confident | 0.06 | 0.27 | 0.01 | 0.03 | 0.08 | 0.19 | 0.01 | 0.27 | -0.03 |
|  | Sleep | 0.16 | 0.16 | 0.13 | 0.15 | 0.19 | 0.1 | 0.16 | 0.18 | -0.01 |
|  | Talkative | 0.14 | 0.22 | 0.11 | 0.09 | 0.14 | 0.11 | 0.12 | 0.22 | -0.07 |
|  | Active | 0.13 | 0.21 | 0.08 | 0.07 | 0.1 | 0.18 | 0.09 | 0.24 | -0.06 |
| QIDS | Sleep | **0.33** | -0.11 | 0.29 | 0.25 | 0.26 | -0.14 | **0.34** | -0.03 | -0.04 |
|  | Sad | **0.59** | -0.08 | **0.73** | **0.47** | **0.44** | -0.19 | **0.67** | 0.03 | -0.25 |
|  | Appetite/weight | **0.41** | -0.06 | **0.37** | **0.30** | **0.33** | -0.17 | **0.43** | -0.01 | -0.07 |
|  | Concentration | **0.55** | -0.13 | **0.52** | **0.39** | **0.42** | -0.23 | **0.57** | -0.04 | -0.14 |
|  | Self-view | **0.55** | -0.1 | **0.61** | **0.41** | **0.41** | -0.21 | **0.60** | 0 | -0.20 |
|  | Suicide | **0.44** | -0.11 | **0.55** | **0.39** | **0.35** | -0.19 | **0.51** | -0.02 | -0.13 |
|  | Interest | **0.47** | -0.11 | **0.55** | **0.37** | **0.39** | -0.21 | **0.54** | -0.02 | -0.14 |
|  | Energy | **0.50** | -0.17 | **0.53** | **0.36** | **0.37** | -0.28 | **0.55** | -0.10 | -0.16 |
|  | Restless | **0.55** | -0.09 | **0.53** | **0.4** | **0.43** | -0.17 | **0.58** | 0.02 | -0.13 |
| GAD-7 | Nervous/anxious | **0.67** | -0.05 | **0.60** | **0.44** | **0.47** | -0.17 | **0.66** | 0.06 | -0.22 |
|  | Control worries | **0.63** | -0.06 | **0.62** | **0.47** | **0.47** | -0.16 | **0.64** | 0.07 | -0.19 |
|  | Worried | **0.64** | -0.04 | **0.61** | **0.45** | **0.46** | -0.14 | **0.63** | 0.08 | -0.21 |
|  | Relaxed | **0.63** | -0.07 | **0.58** | **0.44** | **0.47** | -0.16 | **0.63** | 0.05 | -0.18 |
|  | Restless | **0.51** | 0.03 | **0.48** | **0.4** | **0.39** | -0.06 | **0.51** | 0.13 | -0.13 |
|  | Irritable | **0.57** | 0 | **0.53** | **0.53** | **0.62** | -0.13 | **0.63** | 0.12 | 0.02 |
|  | Afraid | **0.63** | -0.09 | **0.63** | **0.48** | **0.47** | -0.17 | **0.64** | 0.04 | -0.17 |
| EQ-5D | | **-0.55** | 0.22 | **-0.52** | **-0.42** | **-0.46** | **0.37** | **-0.62** | 0.15 | 0.07 |
| Total | ASRM | 0.15 | 0.26 | 0.10 | 0.10 | 0.15 | 0.19 | 0.11 | 0.29 | -0.07 |
|  | QIDS | **0.60** | -0.12 | **0.64** | **0.45** | **0.48** | -0.24 | **0.66** | -0.01 | -0.19 |
|  | GAD-7 | **0.71** | -0.04 | **0.66** | **0.51** | **0.56** | -0.16 | **0.72** | 0.09 | -0.19 |

Bold entries indicate statistically strong associations (Spearman $\left| R \right|\geq0.3$). We used the nine QIDS domains rather than the 16 items, because depression is clinically assessed in this way. Each of the items of the weekly questionnaires is presented as a sentence to participants; we present these as words here to facilitate comparisons.

Exploring MZ items association for each of the three cohorts

In this section we explore the statistical associations of the MZ items and the established weekly questionnaires, for each of the three groups. The results should be contrasted against Table 3 in the manuscript where the data from all three groups was used to compute these relationships.

**Table S9**: Statistical associations (Spearman correlation coefficient) between the averaged MZ over each week and the constituent items of the established weekly questionnaires (ASRM, QIDS, GAD-7, EQ-5D) for BD participants.

|  | | MZ items | | | | | | MZ factors | | |
| --- | --- | --- | --- | --- | --- | --- | --- | --- | --- | --- |
|  |  | Anxious | Elated | Sad | Angry | Irritable | Energetic | negative | positive | irritability |
| ASRM | Happy | 0.02 | **0.36** | -0.03 | 0.03 | 0.09 | 0.22 | -0.02 | **0.33** | -0.01 |
|  | Confident | 0.02 | **0.36** | -0.06 | 0.04 | 0.09 | 0.23 | -0.03 | **0.35** | 0.03 |
|  | Sleep | 0.12 | 0.17 | 0.05 | 0.18 | 0.24 | 0.07 | 0.14 | 0.17 | 0.11 |
|  | Talkative | 0.05 | 0.28 | -0.03 | 0.09 | 0.14 | 0.15 | 0.04 | 0.26 | 0.07 |
|  | Active | 0.03 | 0.29 | 0 | 0.07 | 0.06 | 0.23 | 0.01 | **0.30** | -0.02 |
| QIDS | Sleep | **0.27** | -0.13 | 0.13 | 0.25 | 0.3 | -0.15 | 0.27 | -0.05 | 0.18 |
|  | Sad | **0.48** | -0.23 | **0.71** | **0.47** | **0.42** | -0.23 | 0.63 | -0.09 | -0.16 |
|  | Appetite/weight | **0.32** | -0.17 | 0.13 | 0.21 | 0.28 | -0.2 | **0.30** | -0.10 | 0.09 |
|  | Concentration | **0.53** | **-0.33** | **0.41** | **0.41** | **0.45** | **-0.35** | **0.56** | -0.21 | 0.01 |
|  | Self-view | **0.40** | -0.21 | **0.49** | **0.33** | **0.34** | -0.23 | **0.48** | -0.11 | -0.08 |
|  | Suicide | **0.39** | -0.16 | **0.55** | **0.44** | **0.36** | -0.17 | **0.51** | -0.02 | -0.01 |
|  | Interest | **0.36** | -0.17 | **0.45** | **0.34** | **0.33** | -0.20 | **0.44** | -0.06 | -0.01 |
|  | Energy | **0.41** | -0.3 | **0.41** | **0.31** | **0.33** | **-0.32** | **0.45** | -0.21 | -0.05 |
|  | Restless | **0.45** | -0.21 | **0.41** | **0.39** | **0.44** | -0.18 | **0.51** | -0.06 | 0.08 |
| GAD-7 | Nervous/anxious | **0.62** | -0.25 | **0.42** | **0.41** | **0.44** | -0.26 | **0.57** | -0.13 | -0.03 |
|  | Control worries | **0.58** | -0.22 | **0.53** | **0.47** | **0.45** | -0.20 | **0.59** | -0.07 | -0.06 |
|  | Worried | **0.61** | -0.22 | **0.56** | **0.48** | **0.48** | -0.21 | **0.62** | -0.06 | -0.08 |
|  | Relaxed | **0.60** | -0.24 | **0.47** | **0.44** | **0.48** | -0.26 | **0.61** | -0.11 | -0.02 |
|  | Restless | **0.40** | 0.01 | **0.35** | **0.39** | **0.42** | -0.05 | **0.45** | 0.10 | 0.05 |
|  | Irritable | **0.58** | -0.08 | **0.43** | **0.57** | **0.68** | -0.22 | **0.63** | 0.03 | 0.22 |
|  | Afraid | **0.58** | -0.2 | **0.60** | **0.49** | **0.48** | -0.17 | **0.62** | -0.02 | -0.08 |
| EQ-5D | | **-0.48** | 0.12 | **-0.49** | **-0.46** | **-0.50** | **0.32** | **-0.56** | 0.05 | -0.07 |
| Total | ASRM | 0.08 | **0.33** | 0 | 0.13 | 0.17 | 0.20 | 0.06 | **0.32** | 0.05 |
|  | QIDS | **0.56** | -0.29 | **0.57** | **0.47** | **0.50** | **-0.30** | **0.65** | -0.15 | -0.02 |
|  | GAD-7 | **0.70** | -0.22 | **0.57** | **0.55** | **0.59** | **-0.25** | **0.72** | -0.07 | -0.01 |

Bold entries indicate statistically strong associations (Spearman $\left| R \right|\geq0.3$). We used the nine QIDS domains rather than the 16 items, because depression is clinically assessed in this way. Each of the items of the weekly questionnaires is presented as a sentence to participants; we present these as words here to facilitate comparisons.

**Table S10**: Statistical associations (Spearman correlation coefficient) between the averaged MZ over each week and the constituent items of the established weekly questionnaires (ASRM, QIDS, GAD-7, EQ-5D) for BPD participants.

|  | | MZ items | | | | | | MZ factors | | |
| --- | --- | --- | --- | --- | --- | --- | --- | --- | --- | --- |
|  |  | Anxious | Elated | Sad | Angry | Irritable | Energetic | negative | positive | irritability |
| ASRM | Happy | -0.17 | 0.28 | -0.08 | -0.09 | -0.08 | 0.24 | -0.17 | 0.24 | -0.04 |
|  | Confident | -0.11 | 0.25 | -0.01 | -0.03 | -0.04 | 0.23 | -0.1 | 0.24 | -0.04 |
|  | Sleep | 0.03 | 0.18 | 0.12 | 0.04 | 0.06 | 0.23 | 0.05 | 0.22 | -0.08 |
|  | Talkative | -0.02 | 0.12 | 0.11 | -0.01 | 0 | 0.16 | 0 | 0.15 | -0.11 |
|  | Active | 0 | 0.23 | 0.05 | 0.06 | 0.10 | **0.31** | 0.02 | 0.28 | 0.01 |
| QIDS | Sleep | 0.19 | -0.09 | 0.12 | 0.06 | 0.08 | -0.09 | 0.16 | -0.06 | -0.03 |
|  | Sad | **0.34** | -0.19 | 0.43 | 0.20 | 0.19 | -0.13 | **0.36** | -0.08 | -0.13 |
|  | Appetite/weight | 0.16 | 0.06 | 0.18 | 0.19 | 0.21 | -0.05 | 0.21 | 0.09 | 0.07 |
|  | Concentration | 0.17 | -0.03 | 0.2 | 0.03 | -0.02 | -0.07 | 0.12 | -0.03 | -0.18 |
|  | Self-view | **0.31** | -0.09 | **0.34** | 0.13 | 0.08 | -0.07 | 0.27 | -0.02 | -0.22 |
|  | Suicide | 0.13 | -0.23 | 0.24 | 0.05 | 0.04 | -0.2 | 0.16 | -0.2 | -0.11 |
|  | Interest | 0.21 | -0.23 | 0.24 | 0.03 | 0.04 | -0.21 | 0.19 | -0.18 | -0.13 |
|  | Energy | 0.28 | -0.14 | 0.27 | 0.05 | 0.03 | -0.23 | 0.22 | -0.12 | -0.21 |
|  | Restless | **0.31** | 0.04 | 0.25 | 0.07 | 0.11 | 0.05 | 0.22 | 0.1 | -0.21 |
| GAD-7 | Nervous/anxious | **0.47** | -0.12 | **0.37** | 0.26 | 0.27 | -0.1 | **0.43** | -0.01 | -0.08 |
|  | Control worries | **0.52** | -0.08 | **0.42** | **0.32** | **0.32** | -0.09 | **0.48** | 0.04 | -0.08 |
|  | Worried | **0.48** | -0.03 | **0.35** | 0.26 | 0.27 | -0.09 | **0.41** | 0.05 | -0.07 |
|  | Relaxed | **0.48** | -0.01 | **0.40** | 0.27 | 0.28 | 0.05 | **0.42** | 0.13 | -0.1 |
|  | Restless | **0.38** | 0.17 | 0.24 | 0.17 | 0.19 | 0.12 | 0.27 | 0.23 | -0.11 |
|  | Irritable | 0.25 | 0.07 | 0.21 | **0.46** | **0.54** | 0.03 | **0.40** | 0.18 | **0.41** |
|  | Afraid | **0.48** | -0.09 | **0.39** | **0.35** | **0.37** | -0.18 | **0.50** | 0 | 0.01 |
| EQ-5D | | **-0.46** | 0.32 | -0.31 | -0.2 | -0.2 | 0.36 | -0.4 | 0.26 | 0.07 |
| Total | ASRM | -0.09 | 0.27 | 0.01 | -0.01 | 0.01 | 0.29 | -0.07 | 0.28 | -0.04 |
|  | QIDS | **0.37** | -0.17 | **0.41** | 0.13 | 0.12 | -0.18 | **0.33** | -0.1 | -0.22 |
|  | GAD-7 | **0.56** | -0.01 | **0.44** | **0.39** | **0.42** | -0.04 | **0.54** | 0.13 | 0 |

Bold entries indicate statistically strong associations (Spearman $\left| R \right|\geq0.3$). We used the nine QIDS domains rather than the 16 items, because depression is clinically assessed in this way. Each of the items of the weekly questionnaires is presented as a sentence to participants; we present these as words here to facilitate comparisons.

**Table S11**: Statistical associations (Spearman correlation coefficient) between the averaged MZ over each week and the constituent items of the established weekly questionnaires (ASRM, QIDS, GAD-7, EQ-5D) for HC participants.

|  | | MZ items | | | | | | MZ factors | | |
| --- | --- | --- | --- | --- | --- | --- | --- | --- | --- | --- |
|  |  | Anxious | Elated | Sad | Angry | Irritable | Energetic | negative | positive | irritability |
| ASRM | Happy | 0.07 | 0.15 | 0.08 | 0.08 | 0.09 | 0.17 | 0.03 | 0.19 | -0.06 |
|  | Confident | 0.06 | 0.14 | 0.04 | 0.05 | 0.08 | 0.18 | 0.01 | 0.19 | -0.05 |
|  | Sleep | 0.11 | 0.13 | 0.1 | 0.08 | 0.12 | 0.1 | 0.07 | 0.13 | -0.02 |
|  | Talkative | 0.15 | 0.20 | 0.15 | 0.12 | 0.17 | 0.14 | 0.11 | 0.19 | -0.05 |
|  | Active | 0.08 | 0.10 | 0.04 | 0.05 | 0.05 | 0.14 | 0.03 | 0.15 | -0.05 |
| QIDS | Sleep | 0.03 | -0.08 | 0.05 | 0.07 | 0.09 | 0.04 | 0.04 | -0.01 | 0.08 |
|  | Sad | **0.39** | **0.30** | **0.47** | **0.32** | **0.32** | 0.17 | **0.34** | 0.28 | **-0.30** |
|  | Appetite/weight | 0.14 | 0.03 | 0.1 | 0.05 | 0.13 | -0.07 | 0.14 | 0 | -0.07 |
|  | Concentration | 0.22 | 0.06 | 0.19 | 0.19 | 0.29 | 0.03 | 0.19 | 0.07 | 0.05 |
|  | Self-view | 0.19 | 0.23 | 0.26 | 0.18 | 0.18 | 0.13 | 0.16 | 0.21 | -0.16 |
|  | Suicide | 0.15 | 0.18 | 0.21 | 0.13 | 0.17 | 0.12 | 0.14 | 0.17 | -0.08 |
|  | Interest | 0.18 | 0.11 | 0.18 | 0.16 | 0.15 | 0.03 | 0.18 | 0.09 | -0.08 |
|  | Energy | 0.16 | 0 | 0.13 | 0.09 | 0.16 | -0.09 | 0.17 | -0.03 | -0.04 |
|  | Restless | 0.11 | 0.10 | 0.13 | 0.14 | 0.20 | 0.05 | 0.13 | 0.09 | 0.04 |
| GAD-7 | Nervous/anxious | **0.44** | **0.30** | **0.44** | **0.31** | **0.32** | 0.20 | **0.32** | **0.30** | **-0.32** |
|  | Control worries | 0.27 | **0.31** | **0.34** | 0.25 | 0.24 | 0.25 | 0.16 | **0.32** | -0.27 |
|  | Worried | **0.32** | 0.29 | **0.36** | 0.27 | **0.30** | 0.23 | 0.22 | **0.30** | -0.23 |
|  | Relaxed | **0.33** | 0.19 | **0.33** | 0.29 | **0.33** | 0.15 | 0.29 | 0.21 | -0.13 |
|  | Restless | 0.27 | 0.13 | 0.26 | 0.27 | 0.25 | 0.11 | 0.26 | 0.16 | -0.09 |
|  | Irritable | **0.40** | 0.22 | **0.39** | **0.36** | **0.47** | 0.14 | **0.36** | 0.23 | -0.11 |
|  | Afraid | 0.27 | 0.12 | 0.25 | 0.19 | 0.22 | 0.09 | 0.23 | 0.14 | -0.14 |
| EQ-5D | | -0.20 | 0.27 | -0.08 | -0.08 | -0.13 | 0.40 | **-0.32** | **0.33** | -0.11 |
| Total | ASRM | 0.15 | 0.19 | 0.11 | 0.1 | 0.13 | 0.19 | 0.08 | 0.22 | -0.10 |
|  | QIDS | 0.25 | 0.05 | 0.27 | 0.21 | 0.28 | 0.06 | 0.24 | 0.09 | -0.07 |
|  | GAD-7 | **0.49** | **0.32** | **0.50** | **0.39** | **0.48** | 0.23 | **0.40** | **0.33** | -0.25 |

Bold entries indicate statistically strong associations (Spearman $\left| R \right|\geq0.3$). We used the nine QIDS domains rather than the 16 items, because depression is clinically assessed in this way. Each of the items of the weekly questionnaires is presented as a sentence to participants; we present these as words here to facilitate comparisons.

Collectively, the correlations reported in Tables S9, S10, and S11, are in fairly good agreement. In general, MZ does not correlate well with ASRM either for all the data together (Table 3 in the manuscript) or for the groups individually. MZ in general correlates more strongly with the items of the established questionnaires for BD participants. This might reflect the familiarization of the BD participants with longitudinal self-monitoring on standardized questionnaires (particularly ASRM and QIDS), since this group was primarily recruited from previous studies which involve longitudinal monitoring.

Overall, MZ correlates sufficiently well with established clinical questionnaires for all three groups.
